# Supplementary material for: Acid phosphatase-like proteins, a biogenic amine and leukotriene-binding salivary protein family from the flea Xenopsylla cheopis
Source: Commun Biol. 2023 Dec 18;6:1280. doi: 10.1038/s42003-023-05679-0 (PMC10728186; doi:10.1038/s42003-023-05679-0)
Supplement: Supplementary file 2 — Supplementary information [file 42003_2023_5679_MOESM2_ESM.pdf]

Supplementary figure 1

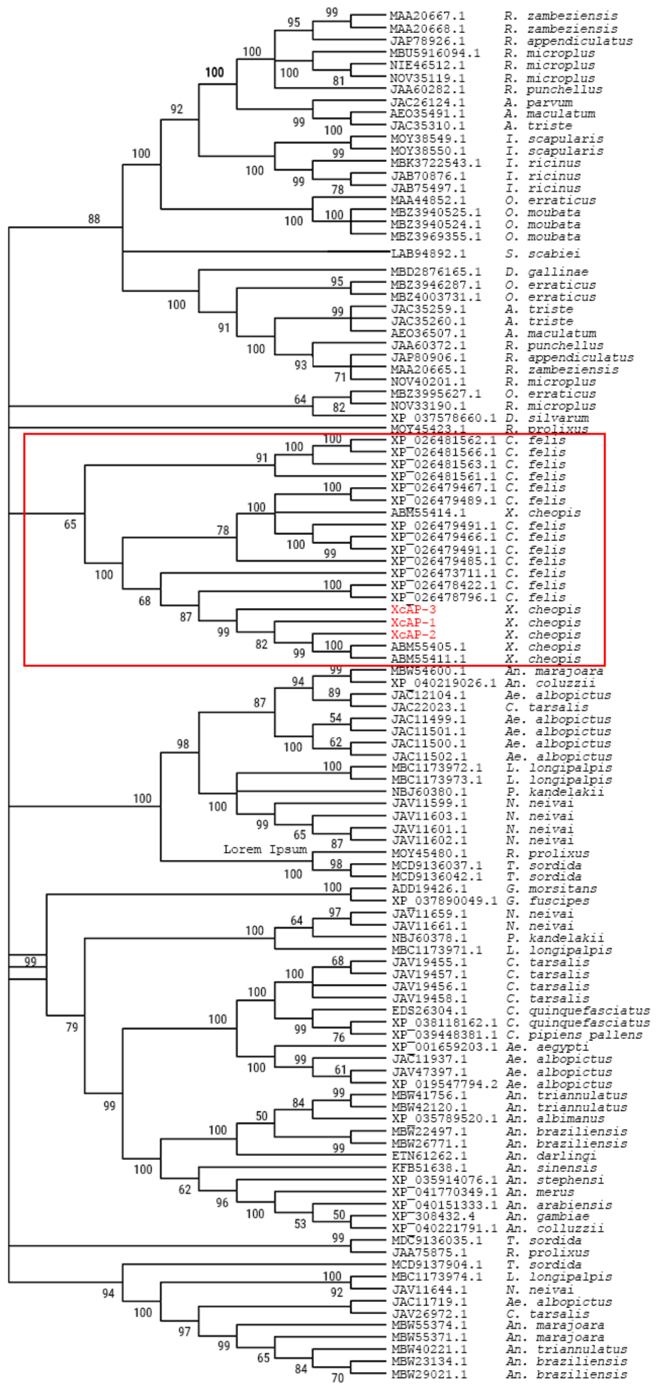


**Supplementary figure 1:** Phylogenetic tree of complete acid phosphatase-like sequences from different blood feeding arthropods including fleas (*X. cheopis* and *C. felis*), mosquitoes (*Culex* sp., *Anopheles* sp., *Aedes* sp.), sand flies (*Phlebotomus kandelakii*, *Nyssomyia neivai* and *Lutzomyia longipalpis*), triatomines (*Triatoma* sp. and *Rhodnius* sp.), ticks (*Dermancentor* sp., *Ornithodoros* sp., *Ixodes* sp., *Rhipicephalus* sp. and *Amblyomma* sp.), flies (*Glossina* sp.) and mites (*Sarcoptes scabiei* and *Dermanyssus gallinae*). The

flea clade and XcAP-1, -2 and -3 are highlighted in red. The number at the base of each branch indicates the concordance between 500 bootstraps replicates.

Supplementary figure 2

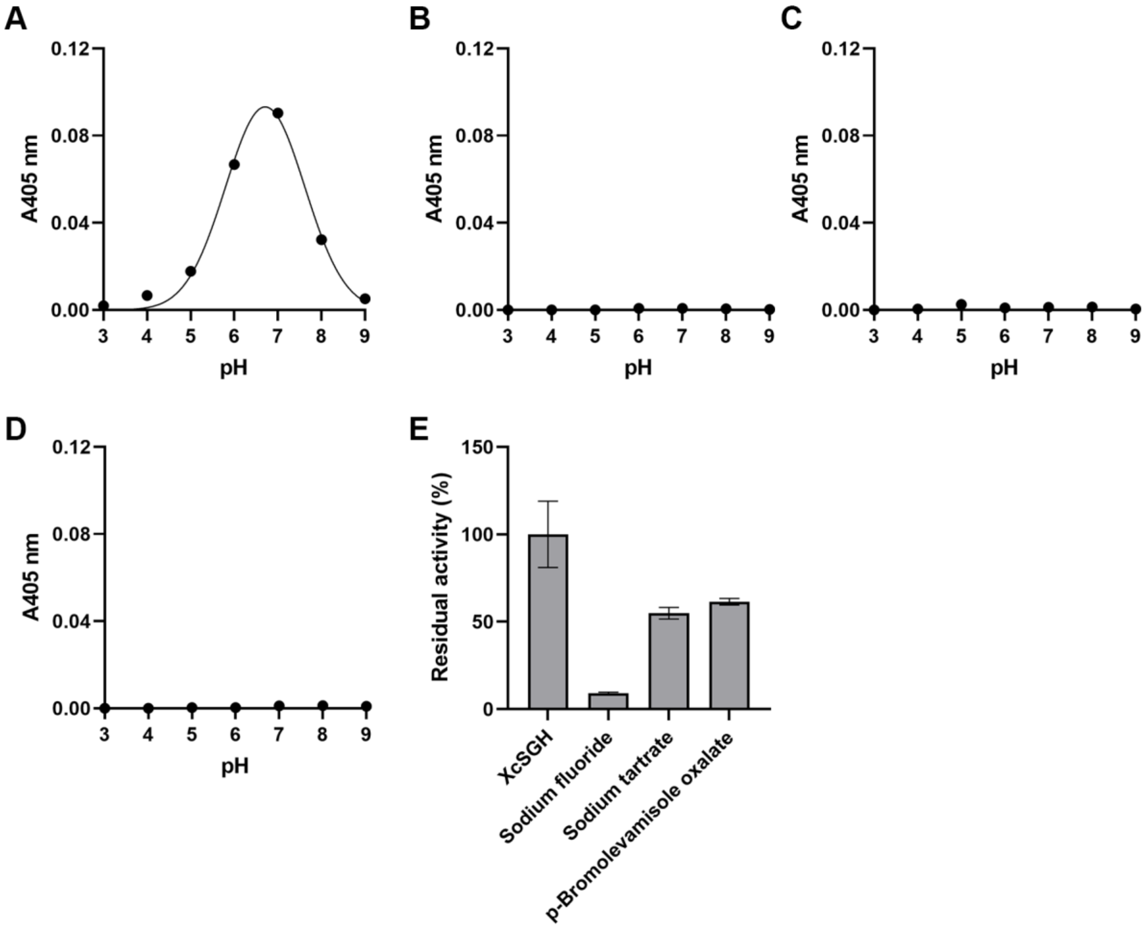


**Supplementary figure 2:** Characterization of the phosphatase activity identified in the salivary glands homogenates of *Xenopsylla cheopis* (XcSGH). **(A)** Determination of the optimum pH of *X. cheopis* SGH (0.01 mg/ml) activity towards p-Nitrophenylphosphate (pNPP)(5 mM). pH curves of recombinant **(B)** XcAP-1, **(C)** XcAP-2 and **(D)** XcAP-3. **(E)** Measurement of the acid phosphatase activity of *X. cheopis* SGH in the presence of different acid and alkaline phosphatase inhibitors (100 µM). Bars represent the residual activity of SGH in the presence of each inhibitor in relation to the SGH without inhibitors. All experiments were performed duplicates.

Supplementary figure 3

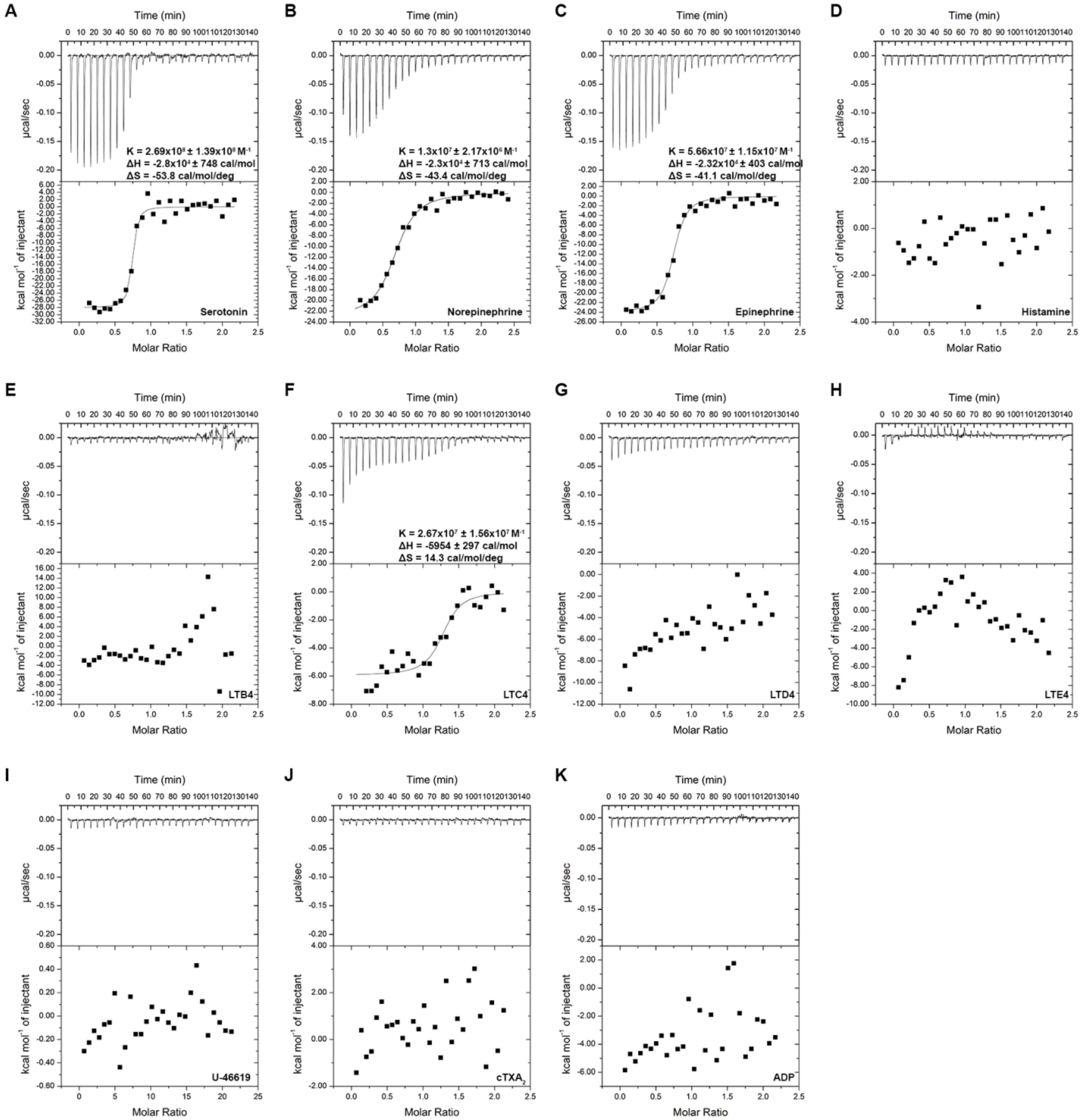


**Supplementary figure 3:** Screening of recombinant XcAP-1 for binding to potential ligands by ITC. The upper plot in each panel shows the measured heat for each injection, while the lower plot shows the injection enthalpies. A single-site binding model was fitted to the data (solid line) and used to estimate the thermodynamic parameters.

Supplementary figure 4

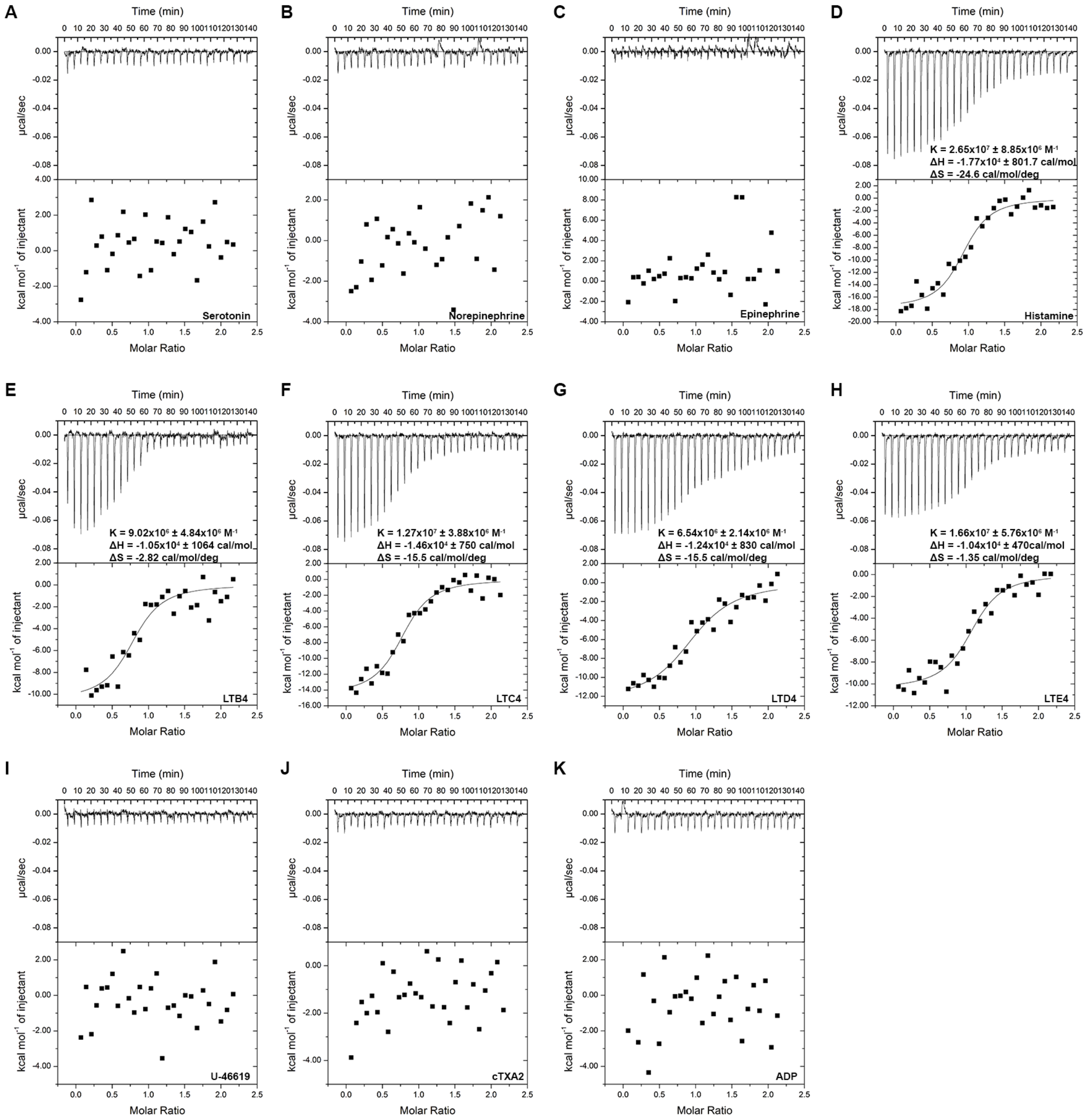


**Supplementary figure 4:** Screening of recombinant XcAP-2 for binding to potential ligands by ITC. The upper curve in each panel shows the measured heats for each injection, while the lower curve shows the enthalpies. A single-site binding model was fitted to the data (solid line) and used to estimate the thermodynamic parameters.

Supplementary figure 5

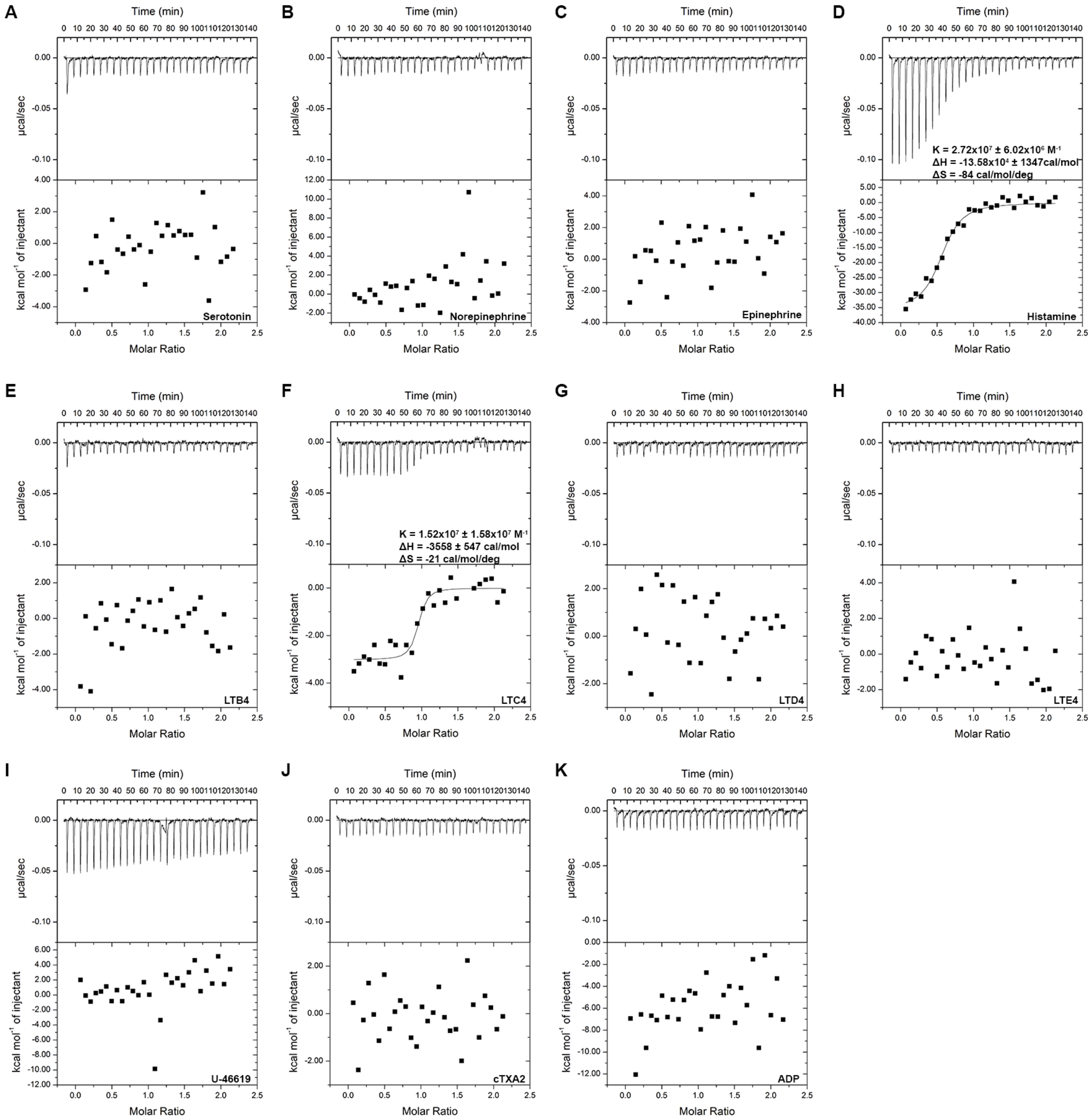


**Supplementary figure 5:** Screening of recombinant XcAP-3 for binding to potential ligands by ITC. The upper curve in each panel shows the measured heat for each injection, while the lower curve shows the enthalpies. A single-site binding model was fitted to the data (solid line) and used to estimate the thermodynamic parameters.

Supplementary figure 6

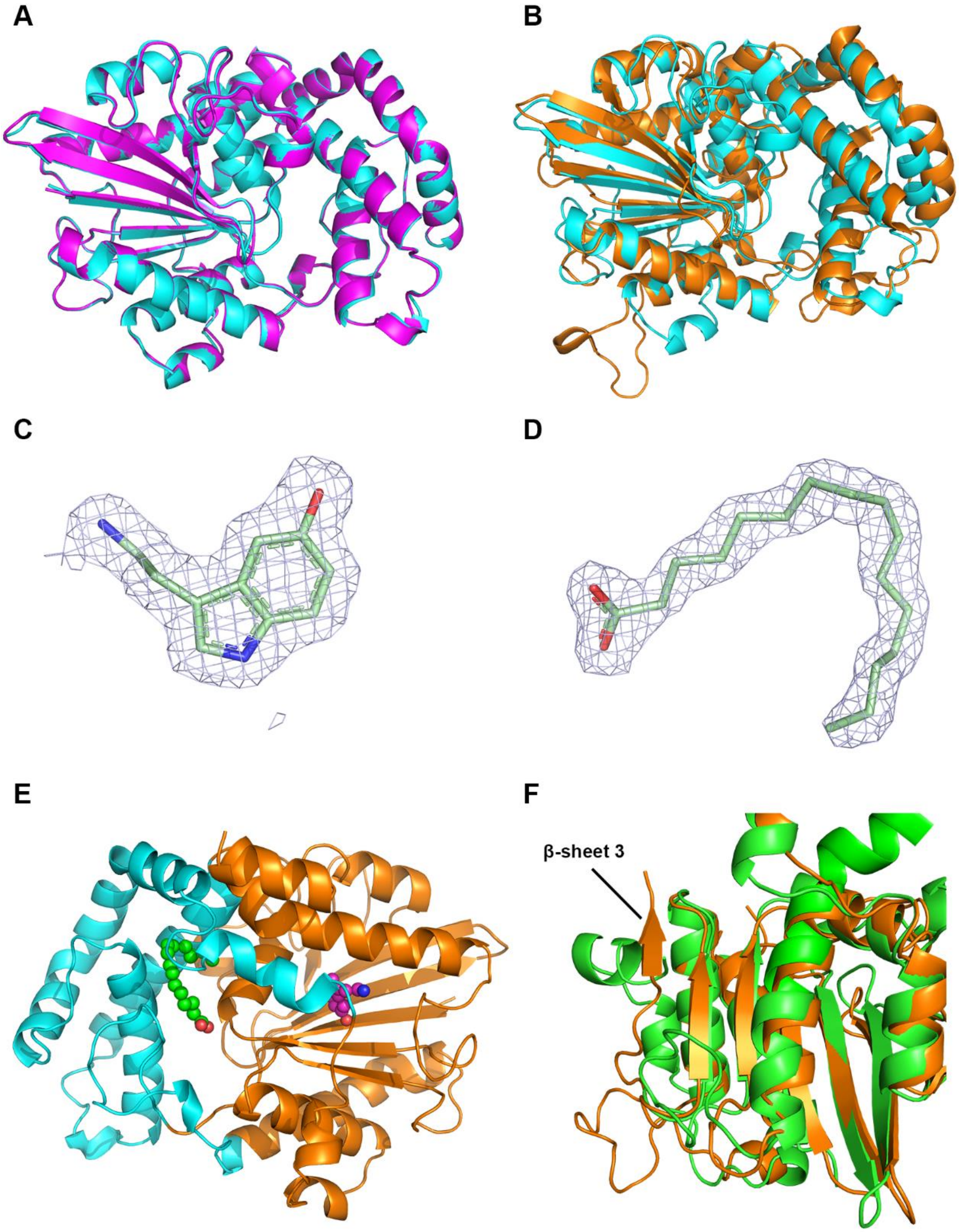


**Supplementary figure 6: (A)** Superposition of the final model of the two XcAP-1:serotonin complexes observed in the asymmetric unit of the unit cell. Each XcAP-1 structure is represented as a cartoon in cyan or magenta. For clarity, the ligands were removed. **(B)** Superposition of XcAP-1 chain A (cyan) with the human prostatic acid phosphatase crystal structure (PDB: 1ND6, orange). **(C)** Serotonin and (**D**) palmitoleic

acid ligands covered by Fo – Fc density map contoured at 1.0 σ. Carbon atoms are shown in green, nitrogen atoms in blue and oxygen atoms in red. **(E)** XcAP-1 in cartoon representation with the α/β-domain colored in orange and the α-domain in cyan. The ligands, serotonin (magenta) and the palmitoleic acid (green) are shown as spheres. **(F)** Comparison of the α/β-domain of XcAP-1 (green) with the rat acid phosphatase (orange, PDB: 1RPA), highlighting the β-sheet 3 missing from XcAP-1.
