# Supplementary material for: Acid phosphatase-like proteins, a biogenic amine and leukotriene-binding salivary protein family from the flea Xenopsylla cheopis
Source: Commun Biol. 2023 Dec 18;6:1280. doi: 10.1038/s42003-023-05679-0 (PMC10728186; doi:10.1038/s42003-023-05679-0)
Supplement: Supplementary file 3 — Description of Supplementary Materials [file 42003_2023_5679_MOESM3_ESM.docx]

**Description of Additional Supplementary Files**

**File name:** Supplementary Data 1

**Description:** Alignment of the RHG and the HD catalytic regions of acid phosphatases sequences from Homo sapiens (1ND6), fleas (X. cheopis and C. felis), mosquitoes (Culex sp., Anopheles sp., Aedes sp.), sand flies (Phlebotomus kandelakii, Nyssomyia neivai and Lutzomyia longipalpis), triatomines (Triatoma sp., Rhodnius sp., and Panstrongylus sp.), ticks (Dermancentor sp., Ornithodoros sp., Ixodes sp., Rhipicephalus sp. and Amblyomma sp.), flies (Tabanus bromius Page 8 of 20 and Glossina sp.) and mites (Sarcoptes scabiei and Dermanyssus gallinae).

**File name:** Supplementary Data 2

**Description:** Numerical source data for graphs and charts presented in the main text.
